# How has nanomedical innovation contributed to the COVID-19 vaccine development?

**DOI:** 10.2217/nnm-2021-0035

**Published:** 2021-05-11

**Authors:** João Paulo Figueiró Longo, Luis Alexandre Muehlmann

**Affiliations:** ^1^Institute of Biological Sciences, University of Brasília, Brasília DF 70910 900, Brazil; ^2^Faculty of Ceilândia, University of Brasília, Brasília DF 72220 900, Brazil

**Keywords:** COVID-19, innovation, lipid nanoparticles, nanomedicine, nanotechnology, vaccine

In the last 5 years, the nanomedicine community has been deeply involved in discussions on its capacity to bring real-world solutions to the market [1]. As a complex and multidisciplinary innovation process, medical nanotechnology is exposed to unique obstacles or barriers that are difficult to address individually. Nonetheless, within this perspective, all participants on this journey, including academics, industrialists and investors, are trying to improve this important discussion for the nanomedicine ecosystem [1,2].

It is not clear when it started, but it is evident that after huge investments in nanobiotechnology in the early 2000s, all of the society expected a large number of nanomedical products to reach the market, benefiting patients who were waiting for new solutions [2]. Extravagant promises presented in project proposals and preclinical results published at the time announced a forthcoming revolution in different fields of the global economy. That expectation, as we know now at the dawn of the third decade of this millennium, was not followed by industrial acceptance of the nanomedical technologies. The aftermath of the numerous R&D projects conducted so far is not exactly frustrating, but it is far from breathtaking [3].

As most of the nanomedical applications have been centered on oncology, this specific field can be taken as an example of what went on in nanomedicine. An important milestone in the evolution of oncological nanomedicine was reached after the publication of results showing that the classical enhanced permeation and retention (EPR) effect, observed in many preclinical studies, is often not observed in clinical conditions [4]. This phenomenon has been proven useful for tumortargeting in different experimental mice tumor models [5,6], so that the great majority of the preclinical studies involving oncological nanomedicines at the beginning of the ‘nanotechnology era’ aimed at exploring the EPR effect [5,6].

Exploring the EPR effect to increase the relative delivery of chemotherapeutic drugs to tumors by nanosystems was a very attractive model to develop a magic bullet for Ehrlich tumor therapy. Many nanomedicine researchers were co-opted by this ideal, but after decades of intense research on this strategy, we are not even close to achieving such a magic bullet. The translation of the preclinical magic bullet candidates from experimental models to patients has encountered important barriers [2,3].

Additionally, other long-promised nanomedicine tools aiming at the reduction of negative side effects, improvement of dosage, safer administration forms and the use of alternative routes of administration [7] have not hit the market as expected. Metselaar and Lammers (2020) [2,8] suggest that exploring all the possibilities of nanotechnology in pharmacotherapy, even those considered as simpler improvements, instead of only looking for revolutionary magic bullets, would bring more acceptance of nanomedicine by industry and investors.

Another point that needs to be considered in this discussion is the number of new approved nanomedicines, which has consistently risen in the last 5 years. Although it is not the number that meets former expectations, it indicates a recent rise in new nanomedicine products launched on the market. In absolute numbers, 50 new nanomedicines entered the market, most of them approved for oncological conditions. Considering clinical trials, which can potentially generate approved products, the percentage of trials for cancer was almost 70% in 2016 and in 2020 this number fell to close to 50%. This can suggest that some nanomedicine research is moving from oncology to other medical applications [2].

Within this context, the nononcological applications usually aim at reducing some drawbacks that are also common to several other pharmacotherapies [7,9]. Of course, all the background obtained in the oncological applications was very important to these new applications. Furthermore, it is possible that the discussion on what went wrong with oncological nanomedicines also brought other benefits from nanoscience and nanotechnology into other biomedical areas [9].

In fact, general theories about innovation always hold that innovations are not created from scratch, but are rather consequences of the constant evolution of previously available knowledge and technologies. In other words, innovation depends upon the recombination of former ideas or the combination of well-known technologies. In terms of technology evolution, the exchange of ideas and data are a fruitful strategy for flourishing innovation in a given ecosystem, such as the nanomedicine environment [10,11].

A current and striking example of this merging of ideas is lipid nanoparticle vehiculation of RNA in vaccines for SARS-CoV-2 [12,13]. Basically, the RNA molecules present in these vaccines need to be delivered to cells where they are translated into antigenic viral proteins. There is, however, a big problem with that: RNAs are extremely fragile and unstable, and readily hydrolyzed by RNAses in the extracellular media, so a free RNA molecule administered into a tissue cannot reach the cytosol of a target cell.

To overcome this intrinsic limitation, researchers encapsulated the RNA molecules in lipid nanocarriers [12,14]. This approach both protects and delivers the RNA. In this way, the half-life of the RNA is increased, and the delivery to the cytosol of target cells is optimized. In addition, it is important to highlight that the overall negative charge of polynucleotides in physiological pH impairs cellular uptake, which is also circumvented by liposomal delivery. Thus, a lipid nanoparticle has different missions in the vaccine strategy [14].

A particularly interesting point in this history is that lipid nanoparticles, especially the liposomes, were not initially designed for that task. They have been intensely investigated for decades, initially as biomimetic membrane models and later on as drug nanocarriers. More recently, these phospholipid vesicles were successfully used to encapsulate siRNA for the treatment of transthyretin amyloidosis, an autoimmune rare disease [15,16]. This was the first biomedical product to be put on the market that uses oligonucleotides as an active drug [2], proving that the protection provided by the lipid nanocarrier is useful and safe. In a retrospective analysis, however, it would be impossible to foresee that the most studied nanostructure carrier in history, the phospholipid nanoparticles, could play such an important role in the management of a future pandemic.

It is always easier to predict something after it happened. This apparently random evolution of a technology and its consequences are quite impossible to design rationally, and we need to be satisfied in understanding how the process occurred in a retrospective way. This apparent loss of control is typical of the whole scientific development process, and can be related to the frustration felt within the nanomedicine ecosystem that we discussed at the beginning of this text. How could one outline the study of biomimetic membranes aiming at the delivery of nucleic acids? New technologies are intended for solving current problems. Not all of them will succeed, but even then, they can be used to solve future problems.

This is basically how science, technology and innovation correlate to each other. Actually, they do not exist as separate entities in the real world, but are rather part of a unique, cyclic process, which has been clearly dissected. Thus, innovation is physiologically connected to basic science, as well as to technology [10]. With regard to nanomedicine specifically, the field is passing through a maturation process, with a strong scientific background that can be used as a tool to improve the effectiveness of the most varied applications in medicine, as well as in other fields. Thus, as we are experiencing a maturation stage [1], some directions cannot be clear yet, but looking at the potential applications of the field, we do believe that it made absolute sense to have invested in nanomedicine over the last few decades.
